# Comprehensive Genome-Wide Association Analysis Reveals the Genetic Basis of Root System Architecture in Soybean

**DOI:** 10.3389/fpls.2020.590740

**Published:** 2020-12-16

**Authors:** Waldiodio Seck, Davoud Torkamaneh, François Belzile

**Affiliations:** ^1^Département de phytologie, Faculté des sciences de l’agriculture et de l’alimentation (FSAA), Université Laval, Quebec, QC, Canada; ^2^Institut de biologie intégrative et des systèmes (IBIS), Université Laval, Quebec, QC, Canada; ^3^Department of Plant Agriculture, University of Guelph, Guelph, ON, Canada

**Keywords:** candidate gene, genome-wide association, phenotypic variation, resilient varieties, rhizoboxes, root system architecture, single-nucleotide polymorphism

## Abstract

Increasing the understanding genetic basis of the variability in root system architecture (RSA) is essential to improve resource-use efficiency in agriculture systems and to develop climate-resilient crop cultivars. Roots being underground, their direct observation and detailed characterization are challenging. Here, were characterized twelve RSA-related traits in a panel of 137 early maturing soybean lines (Canadian soybean core collection) using rhizoboxes and two-dimensional imaging. Significant phenotypic variation (*P* < 0.001) was observed among these lines for different RSA-related traits. This panel was genotyped with 2.18 million genome-wide single-nucleotide polymorphisms (SNPs) using a combination of genotyping-by-sequencing and whole-genome sequencing. A total of 10 quantitative trait locus (QTL) regions were detected for root total length and primary root diameter through a comprehensive genome-wide association study. These QTL regions explained from 15 to 25% of the phenotypic variation and contained two putative candidate genes with homology to genes previously reported to play a role in RSA in other species. These genes can serve to accelerate future efforts aimed to dissect genetic architecture of RSA and breed more resilient varieties.

## Introduction

The root system plays an important role in the acquisition of essential macro and micronutrients and water from the soil and ensures the anchorage of plants (Zhu et al., 2010; Postma et al., 2014; Kochian, 2017; Robinson et al., 2018). Because roots are underground and are so difficult to observe, a little attention has been paid to plant root systems in selection and breeding program. It has been shown that depending on soil composition, the competition in resources capacity (mobile and immobile nutrients, water) can be affected by the shape and spatial configuration of the plant root system known as root system architecture (RSA) (Fitter, 1987; Lynch, 1995; Kochian, 2017). As consequence, many studies showed that RSA was closely correlated with plant yield (Mutava et al., 2015; Liu et al., 2018; Voss-Fels et al., 2018). In recent years, breeders were conscious of the importance of RSA and investigated to better understand the genetic basis of its variation in plant crops.

The RSA is essentially modulated by the growth inhibition of primary root and lateral roots. It can be also modulated by the formation of adventitious roots and root hairs (Malamy, 2005; Waidmann et al., 2020). Therefore, it is generally characterized by measuring numerical variables that describe the size and abundance of components of the root system (e.g., length of roots, number of lateral root number, diameter of roots etc.). However, other measured variables focus on root system structure such as the type and angle of connection between roots (Hodge et al., 2009). The quantification of these RSA-related traits is a greatest challenge faced by research. Previously, roots were extracted and washed to remove the soil for trait measures, such as the destructive technique known as “shovelomics” (Trachsel et al., 2011). More recently, a considerable number of root phenotype approaches *in situ* have been developed.

These approaches known as non-destructive techniques are generally relied on rhizoboxes, transparent enclosures allowing the study of root system development in two-dimensional (2D) using different substrates such as soil or vermiculite (Trachsel et al., 2011). In contrast, soil-free techniques such as hydroponics (Hargreaves et al., 2009; Ayalew et al., 2018; Beyer et al., 2019), aeroponics (Osvald et al., 2001; Lakhiar et al., 2018; Selvaraj et al., 2019), gel plates (Wojciechowski et al., 2009), and growth pouches (Hund et al., 2009; Adu et al., 2014; Adeleke et al., 2019) are used for a better contrast between roots and substrate. Plant RSA is a three-dimensional (3D) structure and phenotyping systems in 2D are limited to quantify all RSA component features. However, new 3D RSA phenotyping is currently developed using sophisticated tomographic techniques such as magnetic resonance imaging (MRI, Jahnke et al., 2009), positron emission tomography (PET, Garbout et al., 2012) X-ray computed tomography (X-ray CT, Mooney et al., 2012; Rogers et al., 2016). Despite the advantages of these approaches in capturing an undisturbed 3D view of the RSA, the phenotyping of a large population remains extremely demanding both in time and cost. These new advances in RSA phenotyping development constitute an important step for genes related to RSA identification.

During the last year, genome-wide association studies (GWAS) provide a power tool for the identification of genes controlling the complex phenotypes such as RSA-related traits (Famoso et al., 2011; Huang et al., 2012; Courtois et al., 2013; Torkamaneh et al., 2020). In Arabidopsis, Meijón et al. (2014) employed GWAS method, using an agar plate phenotyping method, to identify a gene regulating the length of root meristem and taproot. In rice, *DEEPER ROOTING 1* (*DRO1*), an RSA-related gene that controls root growth angle was identified using a 2D phenotyping system in a rhizotron (Uga et al., 2013). Introducing the deep rooting allele at *DRO1* into a cultivar rice having shallow roots resulted in a drought tolerance progeny maintaining high yield under water stress (Uga et al., 2013). A GWAS study in rice (Kadam et al., 2017) has also enabled the identification of a *SCARECROW*/*SHORTROOT* gene, an ortholog of an Arabidopsis gene shown to affect root architecture (Benfey et al., 1993). This rice gene was reported to increase tolerance to a water-deficit stress.

Soybean has many attributes that make it “super crop.” It constitutes an importance source of protein for food and feed. The content of the latter ranged between 38 and 44% of the total dry weight of the seed (Bilyeu et al., 2016). Soybean plant is also an attractive crop due to its ability to fix, with the help of diazotrophic bacteria (rhizobacteria), the atmospheric nitrogen. This leads to a reduction of nitrogen fertilizers uses and adventitious presence, increasing a sustainable agricultural system (Peoples et al., 1995; Herridge et al., 2008; van Hameren et al., 2013). In soybean, mapping of quantitative trait loci (QTL) has enabled the identification of numerous RSA-related traits (Abdel-Haleem et al., 2011; Brensha et al., 2012; Prince et al., 2015). Despite this, there are still too few studies that underly genes within QTL associated with RSA (Brensha et al., 2012; Manavalan et al., 2015; Prince et al., 2015, 2019). However, there is a gap to fill in soybean root literature particularly in the identification and alleles involved in the biological processes and effects on RSA.

Here, a core set of 137 soybean lines that representative of Canadian short-season soybean was phenotyped for RSA-related traits in rhizoboxes. We carried out a GWAS using a catalog of 2.18M SNPs obtained from a combined dataset resulting from both genotyping by sequencing (GBS) and whole-genome sequencing (WGS) to dissect the genetic basis of RSA in soybean.

## Materials and Methods

### Plant Material and Root System Architecture Phenotyping

A set of 137 lines representative of the extent of genetic diversity among short-session soybeans in Canada was used (Supplementary Table 1). Although they range between maturity groups II to 000, most of these belong to MG 0 (Sonah et al., 2015). The soybean seeds (5 for each line) were germinated in Petri dishes (100 mm × 15 mm, standard size) filled with fine vermiculite (0–2 mm). Each germinated plant (3 replicates per line) was then transplanted into a custom-designed rhizobox (40.6 (L) × 25.4 (W) × 1.5 (H) cm; see Supplementary Figure 1). Each rhizobox was filled with stained vermiculite using methylene blue (1.5 g/100 mL) in order to increase the contrast between the root system and the vermiculite. To maintain roots in the dark, the rhizoboxes were covered with white paper. Rhizoboxes were kept at a 45° angle in a greenhouse (26/20°C and 16/8 h day/night) at Université Laval (Supplementary Figure 2). Plants were watered with a mix of minerals and water (Supplementary Table 2). A detailed description of the phenotyping process is illustrated in Supplementary Figure 2. After 10 days of growth, the upper sheet of acrylamide was removed to expose the roots. The root images were taken using a NIKON D3000 camera installed on a tripod maintaining a fixed distance of 35 cm (between the camera and roots). We used the Automatic Root Image Analysis (ARIA) (Pace et al., 2014) software to extract phenotypic data from the images (Supplementary Figure 3). In total, 12 different RSA-related traits were measured from each 2D image including: total length of roots (TLR), length of primary root (LPR), length of secondary roots (LSR), distribution of total root length (DTLR), total number of roots (TNR), median number of roots (Med), maximum number of roots (Max), depth of root system (DRS), width of root system (WRS), surface of root system (SRS), diameter of primary root (DR), surface area of primary root (SAR). A detailed description of these traits measured by ARIA can be found in Supplementary Table 3. Statistical analysis of the phenotypic data, including analysis of variance (ANOVA), frequency distributions and Pearson correlations, of RSA-related trait was performed using R 3.5^1^.

### Genotyping Data

Genotyping of this population was performed through a hybrid approach combining genotyping by sequencing (GBS) and whole-genome sequencing (WGS). In brief, all 137 soybean lines were genotyped through a GBS protocol based on digestion with *Ape*KI (Elshire et al., 2011; Sonah et al., 2013). The SNPs were called using the Fast-GBS pipeline (Torkamaneh et al., 2017) and aligned against the soybean Williams 82 reference genome (Gmax_275_Wm82.a2.v1) (Schmutz et al., 2010). Genotypes were called using a minimal read depth of 2 and loci with less than 80% missing data. These resulted in a catalog of 56K SNPs. Imputation of missing data was first performed on this catalog of 56K GBS-derived SNPs using BEAGLE v4.1 (Browning and Browning, 2016). We then used a reference panel (4.3M SNPs derived from the WGS of 102 Canadian elite soybean lines; Torkamaneh et al., 2018) to impute all missing loci onto the initial catalog of GBS-derived SNPs, again using BEAGLE v4.1 (Browning and Browning, 2016). Among these 102 resequenced lines, 56 were in common with the association panel described above (i.e., >40% overlap). After imputation of missing loci, VCFtools (Danecek et al., 2011) was used to retain SNPs with a minor allele frequency (MAF) ≥0.05 and heterozygosity ≤0.1, thus producing a catalog of 2.18M SNPs.

### Population Structure and Relatedness

In this catalog of 2.18M SNPs, we performed LD-based pruning (*r*^2^ > 0.5) with PLINK (Purcell et al., 2007), to obtain a reduced but uniformly distributed set of 14K markers. The algorithm fastSTRUCTURE (Raj et al., 2014) was used to characterize population structure with the number of tested subpopulations (K) ranging from 1 to 13 and 3 independent runs of runs of each. A python script (“choseek.py”) was used to determine the most likely K value based on the rate of change in LnP between successive K values. To better support the number of subpopulations, we also built a consensus phylogenetic tree (2,000 replicates) using maximum likelihood method based on the Tamura-Nei model implemented in MEGA7 (Kumar et al., 2016) and performed a principal component analysis (PCA) using GAPIT (Lipka et al., 2012). To determine relatedness among individuals, a kinship matrix was calculated using the efficient mixed-model association (EMMA) method (Kang et al., 2008).

### Genome-Wide Association Analysis on Traits Related to Root System Architecture

GWAS analysis was performed on the full set of filtered WGS-derived markers (2.18M SNPs) using the FarmCPU algorithm (Liu et al., 2016) implemented in the rMVP package on Microsoft Open R^2^. To reduce false-positive signals, we included the population of structure matrix (Q) and a Kinship matrix (K) as covariates. A genome-wide significance threshold <0.05 was used to declare significant associations using the false discovery rate (FDR) test of Benjamini and Hochberg (1995). The proportion of phenotypic variance explained by a most significant marker SNP associated was also calculated (Teslovich et al., 2010).

### Candidate Gene Identification

We used a systematic analytical process to identify candidate genes for RSA-related traits. First, we measured LD (D’) between the peak SNP and all markers located within a 2-Mb window (1-Mb on each side) using PLINK (Purcell et al., 2007). The region of interest was defined as extending from the left-and rightmost markers in high LD (D’ ≥ 0.85) with the peak SNP. Genes residing within such haplotype blocks were extracted from Soybase (Grant et al., 2010). We then focused on genes annotated as being involved in root development using gene ontology (GO) terms. In order to provide more information about potential candidate genes, the “Gene expression and protein tools” (ePlant^2^) for soybeans was used to visualize the expression in tissue related to RSA (e.g., roots, root hair, root tip, nodule etc.) [based on the transcriptomic data of Waese et al. (2017)].

Torkamaneh et al. (2018) reported an extensive catalog which included genetic variations established from the WGS data available for a subset of 56 soybean lines. We inspected this catalog to determine if structural or nucleotide variation (within and overlapping the candidate gene) could be causal variants. The predict impact of the nucleotide variants located within genic regions were examined using SnpEff (Cingolani et al., 2012).

## Results

### Phenotypic Variation of Root System Architecture Traits in Soybean

**FIGURE 1:**
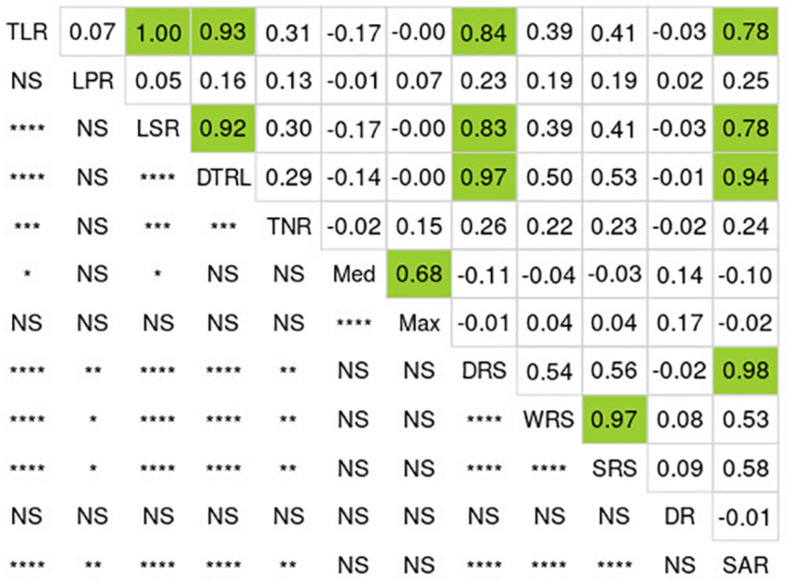
Correlations among RSA-related traits for the 137 soybean lines. Numbers above the diagonal correspond to Pearson’s correlation coefficients (R). Green boxes highlight the values exceeding 0.65. Below the diagonal, we show the degree of significance of the corresponding correlations between traits (****P < 0.0001, ***P < 0.001, **P < 0.01, *P < 0.05, and NS: not significant). TLR, total length of roots; LPR, length of primary root; LSR, length of secondary roots; DTLR, distribution of total root length; TNR, total number of roots; Med, median number of roots; Max, maximum number of roots; DRS, depth of root system; WRS, width of root system; SRS, surface of root system; DR, diameter of primary root; SAR, surface area of primary root.

Wide and significant phenotypic variation was observed among the 137 lines for all RSA-related traits (Table 1). Low coefficients of variation (CV), ranging from 0.1 to 12.1%, were detected among different replications for all RSA-related traits, indicating a high level of reproduciblity of the phenotypic data. Analysis of variance (ANOVA) showed that genotypes are the main source of variation (*P* < 0.001) (Supplementary Table 4). In general, all RSA-related traits followed normal distribution (Shapiro-Wilk test, *p*-value = 0.32) (Figure 1, Supplementary Table 5). Many significant correlations were observed between the 12 traits measured (Figure 1) and three groups of very tightly correlated traits were found (based on *r* > 0.65, *P* < 0.0001). In the first group, we observed that TLR was highly correlated with four other traits: LSR (*r* = 0.99), DTLR (*r* = 0.93), DRS (*r* = 0.84) and SAR (*r* = 0.78). In a second case, WRS was also found to be highly correlated with SRS (*r* = 0.97). In the last group, Med was also correlated with Max (*r* = 0.68). In all of these cases, the most frequently measured RSA-related trait (TLR, WRS and Max) was kept as it was deemed redundant to perform GWAS on all highly correlated traits. Finally, the three remaining traits (LPR, TNR and DR) were not highly correlated to another trait and were each retained for the GWAS.

**TABLE 1:** Summary statistics of the twelve RSA-related traits in the collection of 137 soybean lines.

Traits (unit)^*a*^	Mean ± SE^*b*^	Range	Max/Min	CV (%)^*c*^	Significance^*d*^
TLR (cm)	102.3 ± 4.4	20.0–531.7	26.6	6.4	***
LPR (cm)	13.4 ± 0.5	6.5–19.9	3.1	4.9	***
LSR (cm)	87.7 ± 3.9	3.3–512.2	155.2	5.3	***
DTLR (cm/cm)	0.9 ± 0.1	0.2–2.3	11.5	3.7	***
TNR (#)	59.0 ± 0.7	23.0–123.0	5.3	2.2	***
Med (#)	6.6 ± 0.3	0.7–15.7	22.4	12.1	***
Max (#)	20.0 ± 0.3	7.0–34.9	5.0	6.3	***
DRS (cm)	10.8 ± 0.5	7.4–15.9	2.1	4.8	***
WRS (cm)	8.8 ± 0.7	4.8–14.1	2.9	9.3	***
SRS (cm^2^)	60.9 ± 14.0	15.6–105.7	6.8	0.1	N.S.
DR (cm)	0.128 ± 0.0	0.117–0.132	1.2	1.4	***
SAR (cm^2^)	126.3 ± 1.7	20.4–209.7	10.3	0.3	***

^a^Total length of roots (TLR), length of primary root (LPR), length of secondary roots (LSR), distribution of total root length (DTLR), total number of roots (TNR), median number of roots (Med), maximum number of roots (Max), depth of root system (DRS), width of root system (WRS), surface of root system (SRS), diameter of primary roots (DR), surface area of primary root (SAR). ^b^Standard error (SE). ^c^Coefficient of variation (CV) between replicates. ^d^Significance levels of P-values derived from the ANOVA. ***Indicates a P-value < 0.001, N.S., not significant.

### Genotyping Data and Population Structure

To achieve a dense and exhaustive coverage of the genome, we used a dual genotyping approach combining both GBS and WGS. In a first step, the entire panel was characterized via 56K GBS-derived SNPs. A Canadian soybean reference panel (102 lines, of which 56 were also present in the association panel) of 4.3M WGS-derived SNPs was used to perform imputation of untyped loci in the first catalog. This led to a final catalog of 2.18M SNPs (MAF ≥ 0.05) for a mean density of 1 SNP every 435 bp across the genome.

To characterize population, a subset of 14K LD-pruned SNPs was used. The estimates of the optimum number of subpopulaions (K) ranged between 6 and 9 and trivial differences were observed between these estimates. A phylogenetic tree constructed with the same subset of markers showed seven main branches with bootstrap values ≥50% (Supplementary Figure 5). Similarly, the total variance explained by each principal component (PC) varied between PC1 to PC7. But after PC7, this variance continued to be low and stable. Finally, these results suggested K = 7 as a good estimate of the number of subpopulations within this collection (Supplementary Figure 6).

### Genome-Wide Association of Root System Architecture-Related Traits

**FIGURE 2:**
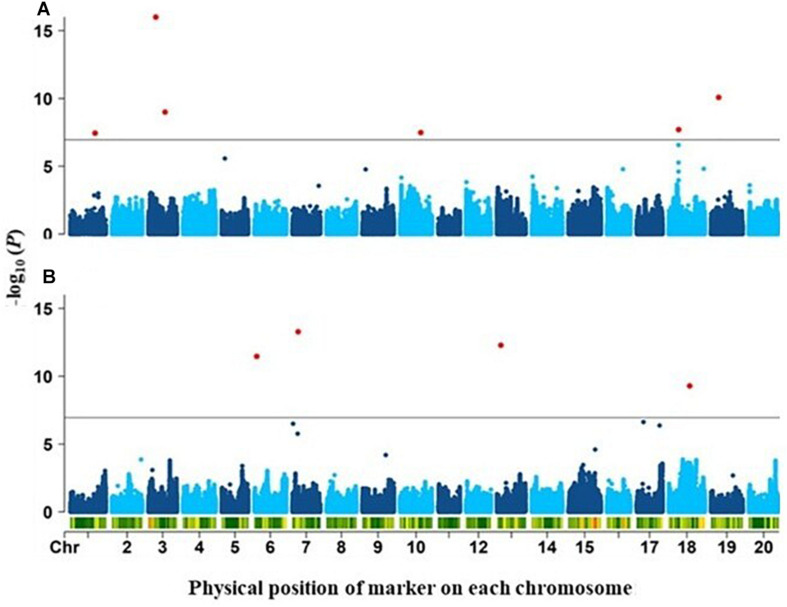
Manhattan plots of the genome-wide association results for **(A)** total length of roots (TLR) and**(B)** diameter of roots (DR). Negative log_10_ (*P*-values) (*y*-axis) describing the strength of the association between each marker and trait are plotted against the physical position of each marker (*x*-axis). The green horizontal line indicates the significance threshold (FDR = 5%) and significant associations are colored in red.

GWAS analysis was performed for twelve RSA-related traits using 2.18M SNPs and the FarmCPU statistical model. To reduce false positive, population structure (Q matrix) and Kinship (K matrix) were incorporated as covariates. In total, 10 SNPs were detected as significantly associated (*p*-value < 1.2e–7; FDR ≤ 0.05) with two RSA-related traits: TLR and DR (Figure 2). Each of these identified a distinct QTL: 6 associated with TLR (*qTLR1* to *qTLR6*) and 4 with DR (*qDR1 to qDR4*) (Table 2). The FDR values associated with these peak SNPs ranged from 0.011 (*qTLR5*) all the way to 2.2 × 10^–10^ (*qTLR2*). While the MAF for three QTLs (*qTLR2, qTLR5* and *qDR3)* was below 0.1, for the seven other QTLs, the MAF ranged between 0.13 and 0.42, such that the estimation of allelic effects (27.7 to 118.4 cm for LTR; 0.018 to 0.037 mm for DR) of the latter QTLs is based on the phenotype of a good number of accessions (≥18). Finally, the phenotypic variance explained (PVE) by these genomic regions varied between 2 and 25% for both traits (TLR and DR). For the four traits (LSR, DTLR, DRS and SAR) highly correlated with TLR, the same six genomic regions were detected in a majority of cases (Supplementary Tables 8, 9). No SNPs were detected as significantly associated with the remaining traits (LPR, TNR, Med, Max, WRS and SRS).

**TABLE 2:** List of quantitative trait loci (QTL) associated with total length of roots (TLR) and diameter of roots (DR) identified in this study.

Trait	Chr	MSS^*a*^ position (bp)	QTL ID	FDR^*b*^ MSS	MAF^*c*^	Effect	PVE^*d*^
TLR (cm)	01	39,473,722	*qTLR1*	1.3e–2	0.35	118.4	0.21
	03	11,872,785	*qTLR2*	2.2e–10	0.09	97.9	0.15
	03	26,421,602	*qTLR3*	7.2e–4	0.18	58.5	0.12
	10	33,249,968	*qTLR4*	1.3e–2	0.22	65.6	0.11
	18	15,820,143	*qTLR5*	1.1e–2	0.07	27.7	0.02
	19	12,124,915	*qTLR6*	9.1e–5	0.35	96.6	0.14
DR (mm)	06	3,828,365	*qDR1*	2.5e–6	0.42	0.021	0.15
	07	8,991,589	*qDR2*	1.1e–7	0.21	0.023	0.25
	13	5,944,486	*qDR3*	5.7e–7	0.09	0.018	0.18
	18	33,584,142	*qDR4*	2.8e–4	0.13	0.037	0.20

*^a^Most significant SNP. ^b^FDR-adjusted p-value. ^c^Minor allele frequency. ^d^Proportion of variation explained by the most significant associated SNP.*

### Root System Architecture-Related Candidate Genes

**FIGURE 3:**
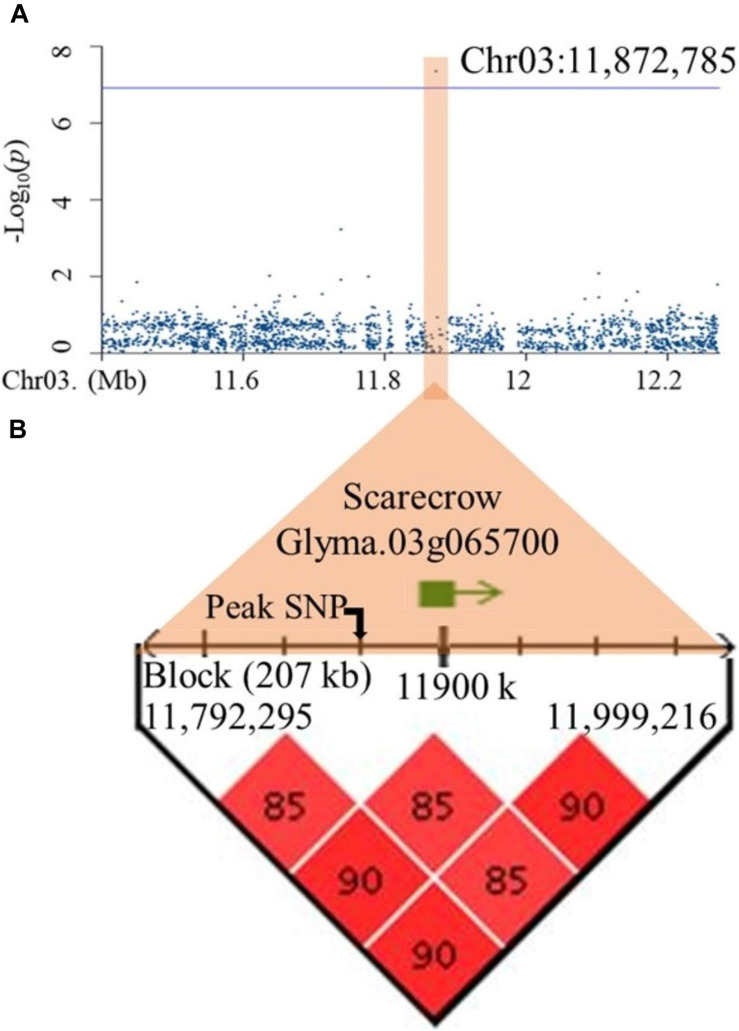
Identification of a candidate gene within the haplotype block containing the peak SNP for qTLR2 on chromosome 3. **(A)** A regional Manhattan plot (1Mb) representing marker-trait associations on chromosome 3. **(B)** Haplotype block including the peak SNP (Chr03: 11,872,785) and a candidate gene (Glyma.03g065700) residing in this block.

To establish a list of candidate genes, regions of interest for all 10 QTLs were defined as spanning from the leftmost to the rightmost marker in high LD (D′ ≥ 0.85) with the peak SNP. All genes residing in whole or in part within one of these ten regions of interest were extracted from SoyBase. These haplotype blocks differed markedly in size, ranging from as little as 1.8 kb (*qDR2*) to as much as 425 kb (*qDR4*) (Supplementary Table 6). Surprisingly, the number of genes per haplotype block was very low across all candidate regions, ranging only between 1 and 3, as exemplified by *qTLR2* for which the haplotype block spanned 207 kb and yet contained a single candidate gene located 24 kb upstream of the peak SNP (Figure 3). The Supplementary Table 7 provides the complete information of these genes including their annotations. On the basis of their annotation and expression, we identified two strong candidate genes, one each for TLR (*Glyma.03g065700*) and DR (*Glyma.07g096000*) (Supplementary Figure 9). In the first case (*qTLR2*), *Glyma.07g096000* encodes a Scarecrow-like protein, a putative transcription factor thought to be involved in root radial patterning and root growth. In addition, transcriptomic data showed that *Glyma.03g065700* was mainly expressed in roots. As for the *qDR2* QTL, the haplotype block spanned 1.8 kb and contained a gene (*Glyma.07g096000*) located 155 bp downstream of the peak SNP (Supplementary Figure 9). This gene encodes an associated receptor protein kinase, a protein thought to play a role in root hair and root tip development. Transcriptomic data also showed that *Glyma.07g096000* was mainly expressed in root hairs and the root tip. For each of these two genes, only one nucleotide variant (SNP) was identified as residing within the coding region and, in each case, was predicted as showing a “low impact” on the protein function.

As structural variants (mainly indels) are typically removed (short indels, <50 bp) or not called (large indels, ≥ 50 bp) when producing SNP catalogs for GWAS, we explored the possibility that structural variants located within these genes could be responsible for the observed association with these phenotypes. After examination of the WGS data for 56 of the lines, we did not identify any indel either within or overlapping with *Glyma.03g065700* or *Glyma.07g096000*. As a result, the phenotypic variation in RSA observed among the 137 lines was not likely due to a loss of function of these candidate genes.

## Discussion

### Significant Phenotypic Variation of Root System Architecture-Related Traits in Soybean

The existence of phenotypic variation within a germplasm pool is necessary for plant breeders to make progress through selection. In the work reported here, we used rhizoboxes to characterize root systems in 2D. In soybean, different phenotyping tools have been used for evaluation of RSA-related traits such as hydroponic system (Liang et al., 2014) or a cone system (Manavalan et al., 2015; Prince et al., 2015, 2019). A distinguishing feature of the use of rhizoboxes is the fact that pictures of the roots system can be taken without any need to first extract the root system from its original growing medium (water or solid substrate).

In this study, the observed variation proved to be very large with the ratio of the maximum: minimum ranging from as little as 1.2:1 (DR) to over 150:1 (LSR) (Table 1) and most traits showing a several-fold difference between minimum and maximum. Such observations are in line with those made in the course of previous work using different sets of germplasm and phenotyping tools. For example, in this work, TLR showed a 26.6-fold difference between the accessions with the shortest and longest root systems. A similarly wide variation for TLR (21.7-fold difference) was reported in the work of Prince et al. (2015). The trait showing the least variation in our work was DR. In two previous studies, root diameter was also reported to vary in a relatively narrow fashion in soybean (1.5 to 2-fold differences; Prince et al., 2015, 2019).

Another characteristic of these phenotypes was their high degree of reproducibility. Even with as few as three replicates, coefficients of variation were <10% in all but one case (Table 1). This suggests that the device used to assess RSA traits (rhizobox) provided a uniform environment and that many of these traits exhibit a relatively high heritability. Again, this result is broadly consistent with what has been in other RSA phenotyping system. For example, in cones filled with turface and sand, Prince et al. (2015) reported a high degree of reproducibility of RSA-related traits, with coefficients of variation ranging from 1 to 7% (four replicates). However, in a hydroponic system, Liang et al. (2014) reported noticeably higher coefficients of variation ranging between 10 and 20%.

### High and Significant Correlations Among Root System Architecture-Related Traits

While the rhizobox and image analysis allowed us to measure 12 different RSA-related traits, we found that many of these traits were highly and significantly correlated. We were able to group the 12 measured traits into 3 groups of very highly correlated (*r* > 0.65, *P* < 0.0001) traits (Figure 1). These results are also in agreement with other reports in the literature. For example, it has been observed that TLR showed a tight correlation with LSR (*r* = 0.82, *P* < 0.01) (Prince et al., 2015). This indicates that much of the length of the root system is contributed by lateral roots at 10 days of growth. However, some RSA-related traits did not show any correlation with others in this study. This was the case for the DR trait. In soybean, similarly, a previous study reported no correlation (*r* < 0.5 in most cases) between root diameter and other root traits (Prince et al., 2015, 2019).

### Genome-Wide Association Using Whole-Genome Data Revealed 10 QTLs Controlling Root System Architecture

In this study, a GWAS was performed using an exhaustive genome-wide set of SNPs (2.18M). To our knowledge, this constitutes the largest marker dataset used to investigate RSA-related traits in soybean. In previous work encompassing both biparaental QTL mapping and GWAS, the number of markers varied between 232 and 38,052 (Liang et al., 2014; Manavalan et al., 2015; Prince et al., 2015, 2019). While a few hundred markers may provide adequate coverage for a biparental QTL map, the resolution is very limited with QTL regions typically spanning many megabases of DNA and containing such a large number of genes that identifying a candidate gene is challenging. In GWAS studies, it is not likely that <40K SNPs will successfully cover the entire genome and capture all haplotypes. As a consequence, genes contributing to the phenotypic variation will evade detection because no marker is in sufficient LD to capture a significant marker-trait association.

Here, we uncovered a total of 10 genomic regions (QTLs) contributing to the length and diameter of the roots. Similarly, in a recent GWAS study on soybean landraces, the four QTL regions detected were for the number of lateral roots and the thickness of roots (Prince et al., 2019). We observed that majority of the QTLs detected in our work had a moderate to small effect on the phenotype, as has been reported in numerous previous studies of RSA traits (Burton et al., 2014; Orman-Ligeza et al., 2014; Rogers and Benfey, 2015).

Despite extensive marker coverage (2.18 M SNPs), a broad and significant degree of phenotypic variation (Table 1) and the fact that the genotype was found to be the most significant source of this variation (Supplementary Figure 4), no significant marker-trait association was found for six of the measured traits. Given the large marker data set and small size of the association panel, it seems unlikely that an insufficient LD between markers and the causal variants is at play. The high degree of reproducibility of the phenotypes (Table 1) and large portion of variance attributed to the genotypes also exclude the hypothesis that the phenotypic data were subject to a large imprecision or environmental effect that could have precluded the identification of a genetic cause to this variation. We cannot, however, exclude the possibility that the underlying genetic determinants of this variation are numerous, each of which makes too small a contribution to be identified confidently. Alternatively, the causal variants may be present at too low a frequency (<5%) and therefore evade detection as markers with low minor allele frequency were not retained. Finally, there could be epistatic interactions between loci that preclude the identification of the individual loci.

### Putative Candidate Genes for Root System Architecture-Associated QTL

In this work, we considered genes to be candidate causal genes if three conditions were met: (1) they were residing in the same haplotype block containing the peak SNP associated with the RSA-related trait (2) their GO annotation was suggestive of a possible role in root development and (3) they were expressed in at least one root-related tissue/organ such as roots, root hairs, nodule, root tip etc. These are admittedly strict definitions as these exclude, for example, cases where a causal gene is of unknown function or where the causal variant is associated with a regulatory region that need not be in close physical proximity to the gene it controls. A complete list of all genes located in the QTL regions surrounding the peak SNP have nonetheless been provided in Supplementary Table 7.

At the *qTLR2* locus, only one gene (*Glyma.03g065700*) was located within the 207 kb haplotype block containing the peak SNP associated with TLR. This gene is annotated as a putative ortholog of the Arabidopsis *SCARECROW* (*SCR*)/*SHORT-ROOT* (*SHR*) family of genes. The transcription factor *SHR*, in Arabidopsis root, plays a key role in the activity of stem cell and controls the transcription of *SCR* regulating the endodermal specification. The mutations of these genes can be manifested by phenotypes with short roots. Also, in rice, both *OsSCR1* and *OsSHR1* are known to control the division of the epidermis-endodermis initial cells (Kamiya et al., 2003; Cui et al., 2007; Mai et al., 2014; Henry et al., 2017).

A single gene (*Glyma.07g096000*) was located in *qDR2* region associated with DR. This gene encodes a receptor-like protein kinase (RLK) known to regulate plant root growth and development in Arabidopsis (Shiu and Bleecker, 2001). The homolog of *Glyma.07g096000* in Arabidopsis shows no direct effect on the diameter of the roots but plays in different aspects in the development of roots in particularly root development and root tip (Racolta et al., 2014; Wei and Li, 2018). Therefore, we believe that this gene could also affect diameter of roots.

## Data Availability Statement

The original contributions presented in the study are publicly available. This data can be found here: https://figshare.com/s/2eb426e0bb988b19e6ca/https://doi.org/10.6084/m9.figshare.12982886

## Author Contributions

DT and FB conceptualized the research project. WS conducted the phenotyping and analyzed data. WS, DT, and FB interpreted all phenotyping and genotyping data, wrote and approved the manuscript.

## Conflict of Interest

The authors declare that the research was conducted in the absence of any commercial or financial relationships that could be construed as a potential conflict of interest.

## Footnotes

1 https://cran.r-project.org/bin/windows/base/old/3.5.0/

2 http://bar.utoronto.ca/eplant/
