# Supplementary material for: Comprehensive Genome-Wide Association Analysis Reveals the Genetic Basis of Root System Architecture in Soybean
Source: Front Plant Sci. 2020 Dec 16;11:590740. doi: 10.3389/fpls.2020.590740 (PMC7772222; doi:10.3389/fpls.2020.590740)
Supplement: Supplementary file 1 [file Data_Sheet_1.docx]

Supplementary Material

# Supplementary Figures and Tables

## Supplementary Figures


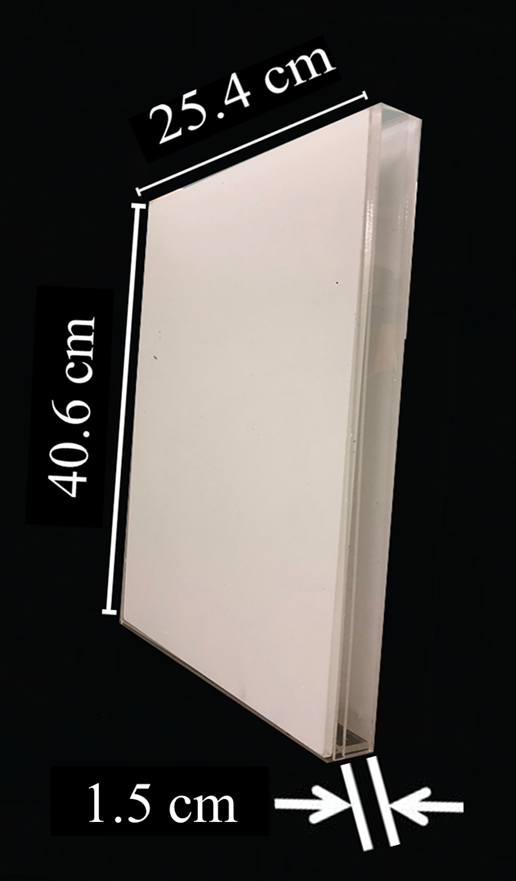


**Supplementary Figure 1**. Description of the rhizobox growing system. The rhizobox encloses two separated acrylicic plates covered with a paper in order to create a shadowed condition for roots. Between the plates, a substrat (e.g. vermiculite) is generally accessible. Plant can developp and the development of its root system can be observed. This system allows clean roots to be harvested and analysis of the two-dimensional RSA.


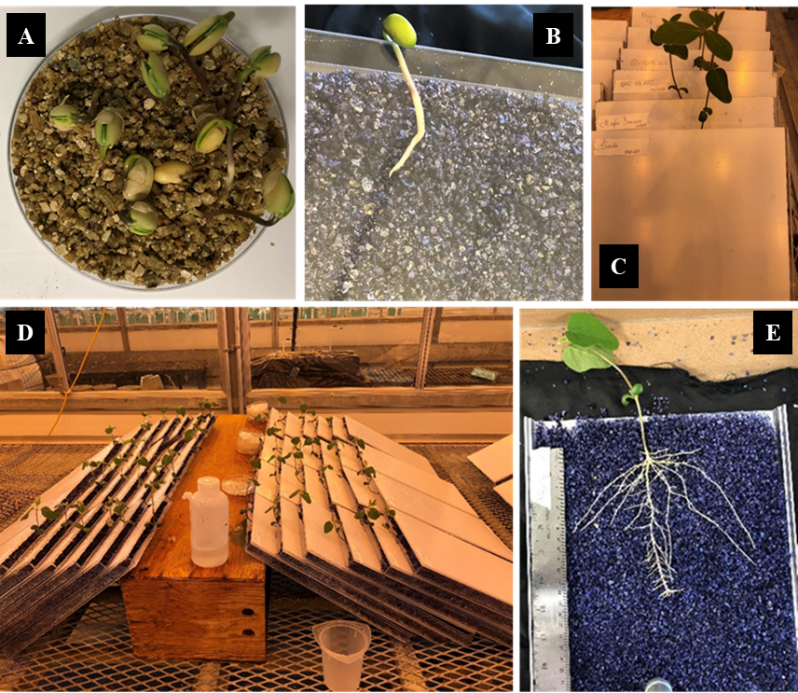


**Supplementary Figure 2**. Root system phenotyping system. (A) Soybean seeds were germinated on vermiculite in a Petri dish. (B) A single germinated soybean seed was positioned at the top of a 1-cm layer of vermiculite enclosed within a rhizobox. (C) Rhizoboxes covered by with paper to find shade for roots. (D) Rhizoboxes were stacked at a 45° angle in a greenhouse. (E) Root system development inside the rhizobox was captured after seven days of growth. The vermiculite was stained with methylene blue to provide good contrast with the root system.


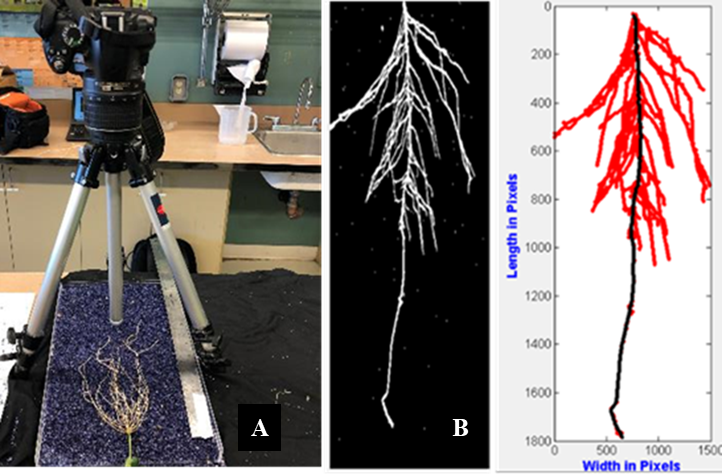


**Supplementary Figure 3.** Root system image acquisition method analysis**. (A)** Imaging root system using tripod camera and cm ruler for scale. **(B)** Automated identification of primary and secondary roots in root system using automatic root image analysis (ARIA) software (Pace et al., 2014).


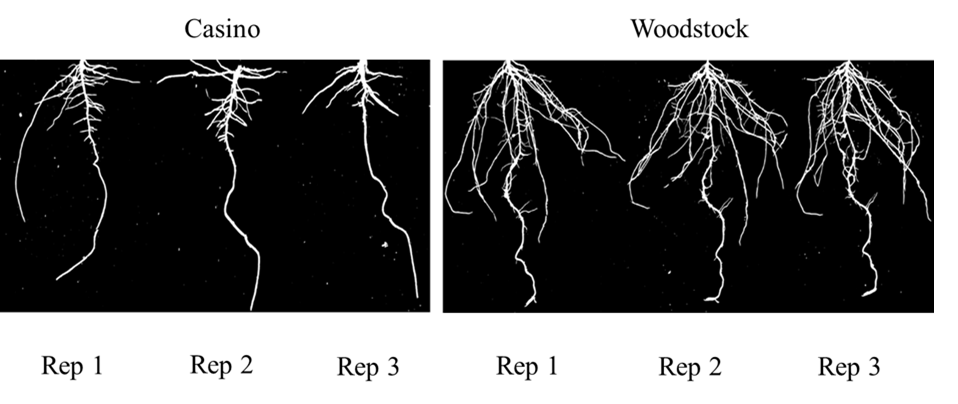


**Supplementary Figure 4**. Root system architecture as observed after ten days of growth in rhizoboxes. Pre-germinated seeds were deposited on the surface of a 1-cm layer of vermiculite tainted with blue dye and enclosed between two sheets of acrylic. After ten days of growth, the top sheet of acrylic was removed, and the root systems were photographed.


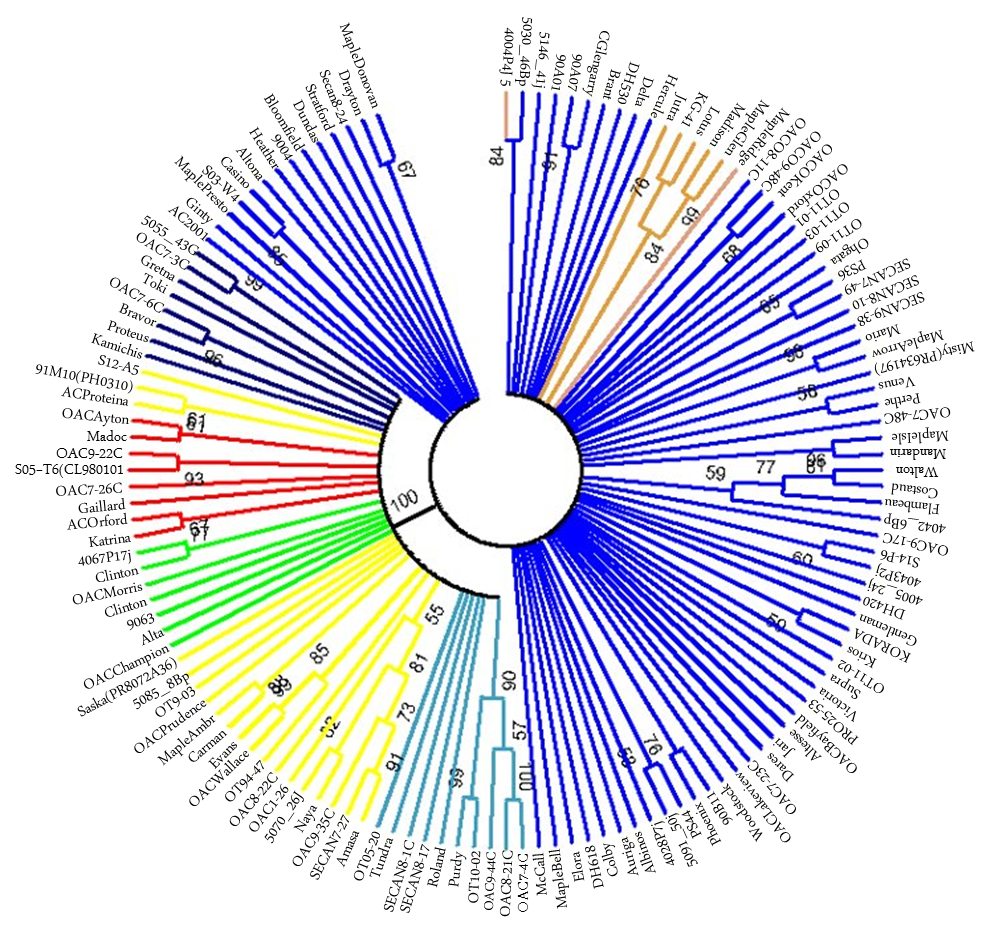


**Supplementary Figure 5.** Bootstrap consensus phylogenetic tree (2000 replicates) constructed using a set of 14K markers and a core set of 137 Canadian soybean lines. Each colour represents a subgroup and seven subgroups were found in total.


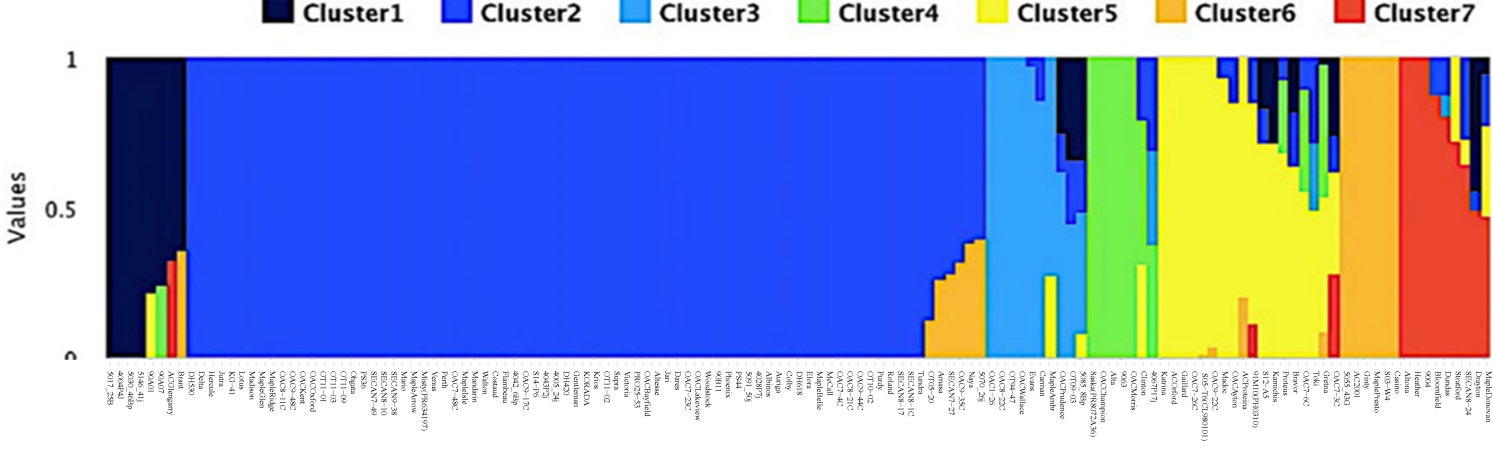


**Supplementary Figure 6.** Population structure in a core set of 137 Canadian soybean lines. Each single vertical line represents each line from 1 to 137. Each color represents one cluster.


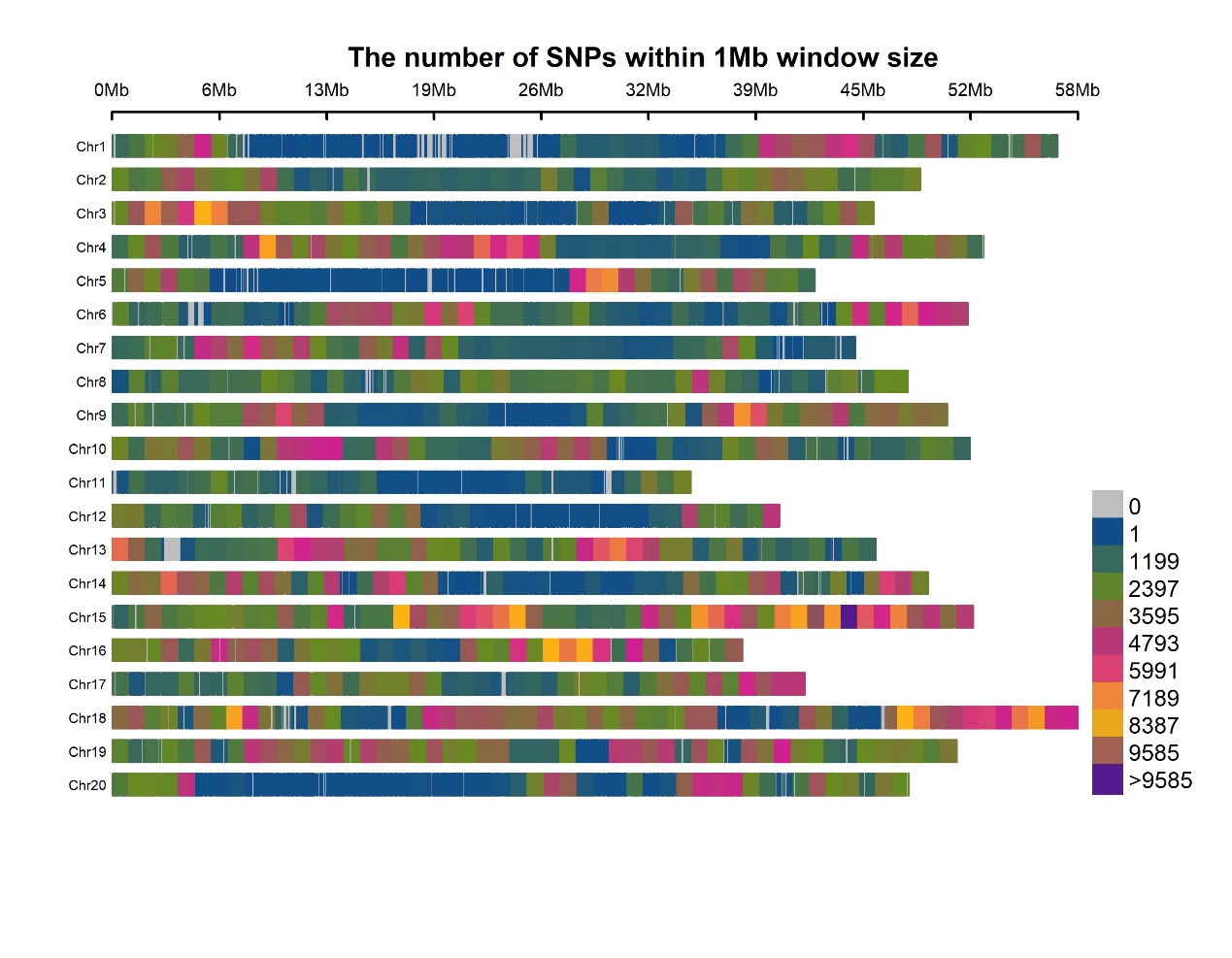


**Supplementary Figure 7.** Distribution of SNP markers across the soybean genome.


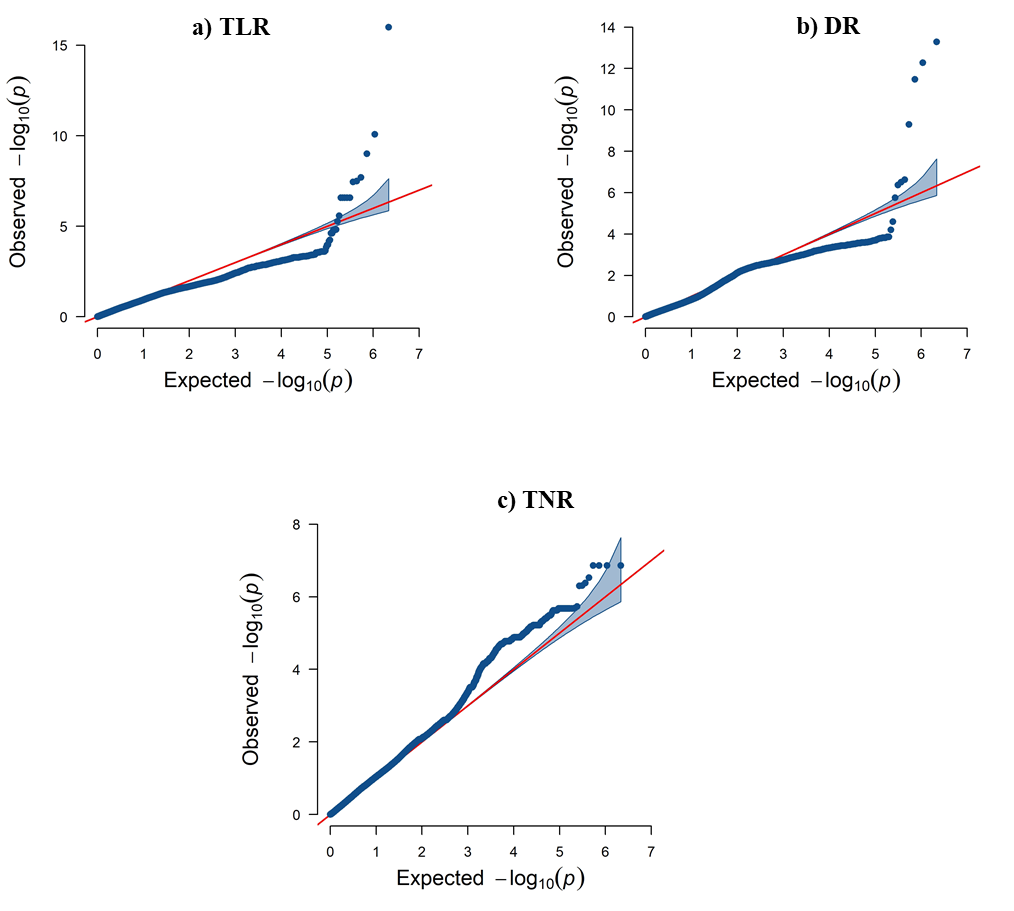


**Supplementary Figure 8:** Quantile-quantile (Q-Q) plot of p-values for the association between SNP markers (blue dots) and a) Total length of roots (TLR), b) Diameter of roots (DR) related-traits using fixed and random model circulating probability unification (FarmCPU) (Liu et al. 2016) in a panel of 137 soybean lines. The y-axis is the observed negative base 10 logarithm of the p-values, and the x-axis is the expected observed negative base 10 logarithm of the p-values under the assumption that the p-values follow a uniform (0,1) distribution. The dotted lines show the 95% confidence interval for the Q-Q plot under the null hypothesis of no association between the SNP and the trait.

;
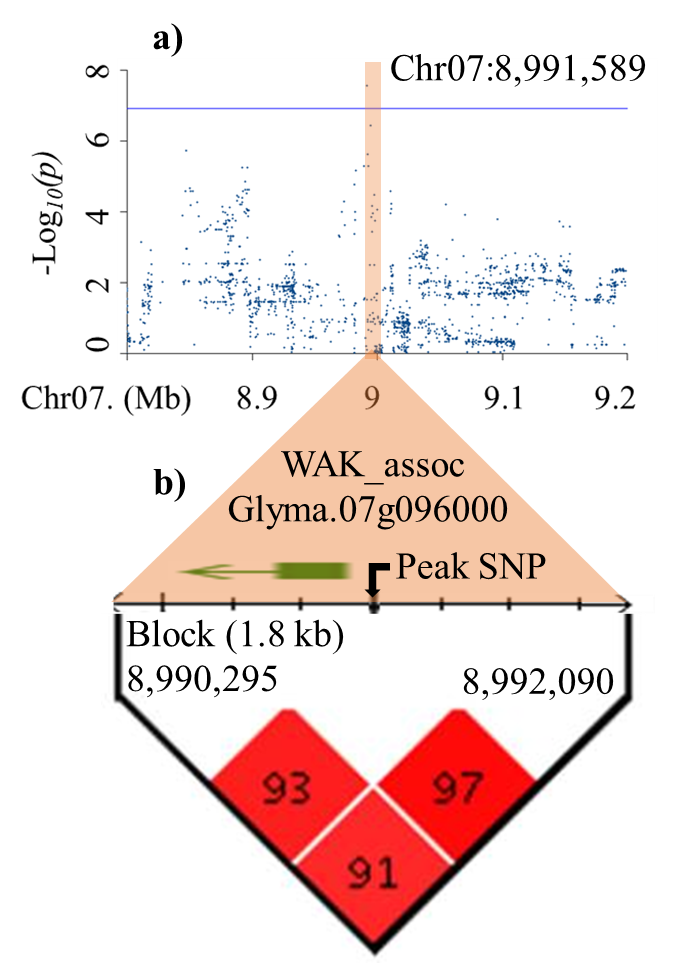


**Supplementary Figure 9:** Identification of a candidate gene within the haplotype block for *qDR2* on chromosome 7. (a) A regional Manhattan plot (1Mb) representing marker-trait associations on chromosome 7. (b) Haplotype block including the peak SNP (Chr07: 8,991,589) and a candidate gene (*Glyma.07g096000*) residing in this block.

## Supplementary Table

**Supplementary Table 1**. List of soybean genotypes (MG0) used in this study with all phenotypic values.

| **Genotype** | **TLR** | **LPR** | **LSR** | **DTLR** | **TNR** | **Med** | **Max** | **DRS** | **WRS** | **RS** | **DR** | **SAR** |
| --- | --- | --- | --- | --- | --- | --- | --- | --- | --- | --- | --- | --- |
| 4004P4J | 71.1 | 13.6 | 62.4 | 0.9 | 24.3 | 7.3 | 14.3 | 10.6 | 10.7 | 82.0 | 0.1290 | 120.1 |
| 4005_24j | 122.4 | 13.8 | 105.2 | 1.2 | 29.0 | 4.7 | 12.0 | 12.1 | 11.2 | 91.5 | 0.1243 | 154.4 |
| 4028P7j | 47.3 | 12.1 | 32.2 | 0.7 | 33.0 | 4.3 | 20.0 | 9.1 | 5.8 | 29.1 | 0.1300 | 90.2 |
| 4042_6Bp | 76.8 | 16.0 | 61.8 | 1.0 | 34.3 | 5.3 | 19.0 | 11.0 | 9.0 | 61.9 | 0.1295 | 131.4 |
| 4043P2j | 42.5 | 14.4 | 24.0 | 0.7 | 35.0 | 6.7 | 18.0 | 9.0 | 7.9 | 50.3 | 0.1262 | 84.2 |
| 4067P17j | 80.1 | 14.9 | 66.2 | 1.0 | 36.0 | 9.0 | 19.3 | 11.1 | 8.5 | 57.6 | 0.1291 | 136.9 |
| 5017_25B | 64.2 | 13.8 | 56.2 | 0.8 | 38.0 | 2.0 | 13.0 | 9.9 | 9.4 | 66.4 | 0.1295 | 107.2 |
| 5030_46Bp | 74.1 | 12.8 | 66.3 | 0.9 | 39.3 | 8.8 | 22.0 | 10.8 | 8.4 | 57.4 | 0.1269 | 125.0 |
| 5055_43G | 66.1 | 12.4 | 55.6 | 0.9 | 40.0 | 11.3 | 22.0 | 10.2 | 5.8 | 32.2 | 0.1238 | 115.9 |
| 5070_26j | 137.3 | 7.8 | 126.0 | 1.3 | 40.3 | 7.0 | 15.7 | 12.4 | 8.3 | 57.2 | 0.1272 | 158.5 |
| 5085_8Bp | 214.1 | 15.7 | 193.2 | 1.4 | 40.7 | 3.7 | 20.0 | 13.2 | 10.8 | 83.4 | 0.1296 | 178.1 |
| 5091_50j | 91.3 | 13.2 | 74.8 | 1.1 | 42.0 | 4.7 | 16.3 | 11.4 | 8.0 | 50.5 | 0.1272 | 143.2 |
| 5146_41j | 138.8 | 11.1 | 124.2 | 1.3 | 42.7 | 3.0 | 14.7 | 12.4 | 7.3 | 45.1 | 0.1292 | 162.5 |
| 9004 | 23.7 | 11.1 | 9.9 | 0.4 | 42.7 | 4.3 | 16.4 | 9.0 | 7.5 | 20.5 | 0.1288 | 58.9 |
| 9063 | 57.9 | 15.9 | 38.3 | 0.8 | 43.3 | 3.3 | 18.0 | 9.8 | 8.1 | 52.2 | 0.1278 | 102.1 |
| 90A01 | 104.1 | 13.5 | 87.4 | 1.1 | 44.7 | 8.3 | 22.0 | 11.6 | 7.1 | 44.7 | 0.1270 | 147.0 |
| 90A07 | 59.7 | 11.9 | 53.6 | 0.8 | 45.0 | 13.7 | 29.7 | 9.8 | 6.6 | 35.2 | 0.1268 | 104.6 |
| 90B11 | 72.0 | 11.2 | 65.7 | 0.9 | 45.7 | 1.3 | 8.3 | 10.7 | 8.0 | 51.2 | 0.1281 | 124.7 |
| 91M10 | 64.7 | 11.9 | 58.5 | 0.8 | 46.0 | 6.3 | 21.3 | 10.0 | 7.7 | 49.7 | 0.1295 | 113.2 |
| AC2001 | 66.1 | 12.9 | 55.1 | 0.9 | 46.0 | 9.0 | 18.0 | 10.3 | 7.4 | 45.2 | 0.1286 | 116.7 |
| ACGlengarry | 36.1 | 10.1 | 23.3 | 0.6 | 46.0 | 2.3 | 14.3 | 8.3 | 6.1 | 33.4 | 0.1291 | 66.5 |
| ACOrford | 45.2 | 15.9 | 26.3 | 0.7 | 46.3 | 2.3 | 18.1 | 9.1 | 8.7 | 59.0 | 0.1286 | 89.5 |
| ACProteina | 103.8 | 15.2 | 85.3 | 1.1 | 47.3 | 5.5 | 20.7 | 11.5 | 6.9 | 40.5 | 0.1301 | 145.7 |
| Albinos | 29.7 | 7.5 | 19.5 | 0.6 | 48.0 | 5.3 | 16.3 | 8.2 | 8.5 | 57.8 | 0.1282 | 66.3 |
| Alta | 47.4 | 12.2 | 32.3 | 0.7 | 48.3 | 4.7 | 17.3 | 9.2 | 7.4 | 47.1 | 0.1294 | 90.7 |
| Altesse | 44.8 | 10.1 | 31.7 | 0.7 | 48.3 | 7.0 | 16.3 | 9.1 | 8.2 | 52.9 | 0.1272 | 87.0 |
| Altona | 38.8 | 9.3 | 25.5 | 0.6 | 48.3 | 6.0 | 22.7 | 8.4 | 6.6 | 35.5 | 0.1267 | 70.0 |
| Amasa | 40.2 | 13.3 | 22.9 | 0.6 | 48.7 | 10.0 | 25.7 | 8.6 | 6.6 | 37.1 | 0.1304 | 73.6 |
| Auriga | 79.5 | 16.3 | 64.1 | 1.0 | 48.7 | 13.0 | 31.3 | 11.1 | 8.2 | 54.5 | 0.1289 | 133.9 |
| Bloomfield | 74.4 | 11.4 | 67.9 | 1.0 | 48.7 | 5.3 | 19.7 | 10.9 | 9.6 | 70.2 | 0.1276 | 125.1 |
| Brant | 61.1 | 16.1 | 50.8 | 0.8 | 48.7 | 7.3 | 20.3 | 9.9 | 9.1 | 62.7 | 0.1290 | 105.8 |
| Bravor | 125.2 | 13.4 | 108.4 | 1.2 | 49.0 | 1.0 | 11.0 | 12.1 | 9.3 | 64.6 | 0.1289 | 154.9 |
| Carman | 68.5 | 15.6 | 54.7 | 0.9 | 50.0 | 3.7 | 14.0 | 10.4 | 8.3 | 55.1 | 0.1289 | 117.6 |
| Casino | 21.2 | 14.2 | 4.3 | 0.4 | 50.0 | 8.7 | 25.3 | 8.6 | 7.8 | 17.0 | 0.1263 | 21.0 |
| Clinton | 62.4 | 11.6 | 56.6 | 0.8 | 50.0 | 5.7 | 17.3 | 9.9 | 7.0 | 43.6 | 0.1281 | 106.4 |
| Colby | 33.4 | 10.2 | 20.5 | 0.6 | 50.7 | 8.0 | 22.3 | 8.3 | 6.7 | 37.2 | 0.1257 | 66.5 |
| Costaud | 120.9 | 11.9 | 105.6 | 1.2 | 51.0 | 5.3 | 19.3 | 12.1 | 9.2 | 63.4 | 0.1269 | 153.6 |
| DH420 | 156.3 | 11.3 | 141.6 | 1.3 | 51.0 | 7.7 | 19.0 | 12.7 | 12.6 | 102.2 | 0.1274 | 164.6 |
| DH530 | 49.6 | 13.8 | 32.8 | 0.7 | 51.3 | 5.0 | 16.7 | 9.2 | 6.9 | 40.8 | 0.1293 | 95.3 |
| DH618 | 96.7 | 12.4 | 81.1 | 1.1 | 51.3 | 4.7 | 22.5 | 11.4 | 7.9 | 50.2 | 0.1258 | 144.6 |
| Dares | 90.9 | 12.2 | 75.4 | 1.0 | 51.7 | 6.7 | 28.3 | 11.3 | 8.9 | 60.6 | 0.1261 | 143.1 |
| Delta | 78.9 | 10.0 | 69.8 | 1.0 | 51.7 | 15.2 | 29.0 | 11.0 | 10.9 | 84.6 | 0.1310 | 132.1 |
| Drayton | 71.0 | 11.2 | 61.6 | 0.9 | 52.3 | 10.0 | 20.0 | 10.6 | 8.8 | 60.4 | 0.1278 | 119.3 |
| Dundas | 24.9 | 8.4 | 13.8 | 0.5 | 52.3 | 5.0 | 19.7 | 8.2 | 8.9 | 61.1 | 0.1309 | 59.7 |
| Elora | 68.8 | 12.3 | 58.4 | 0.9 | 52.3 | 8.3 | 20.7 | 10.5 | 8.3 | 55.7 | 0.1295 | 118.1 |
| Evans | 104.3 | 16.1 | 84.9 | 1.1 | 52.3 | 6.0 | 22.0 | 11.6 | 8.8 | 60.0 | 0.1295 | 147.2 |
| Flambeau | 52.0 | 11.2 | 37.8 | 0.8 | 52.7 | 9.7 | 20.7 | 9.3 | 8.3 | 56.2 | 0.1281 | 98.4 |
| Gaillard | 96.5 | 15.7 | 77.6 | 1.1 | 52.7 | 14.7 | 28.3 | 11.4 | 8.8 | 60.1 | 0.1266 | 144.4 |
| Gentleman | 69.9 | 16.5 | 55.2 | 0.9 | 53.0 | 6.7 | 18.3 | 10.5 | 9.1 | 62.7 | 0.1287 | 119.2 |
| Ginty | 86.9 | 13.3 | 70.3 | 1.0 | 53.3 | 8.0 | 19.3 | 11.3 | 10.9 | 83.5 | 0.1269 | 141.5 |
| Gretna | 47.1 | 14.2 | 30.0 | 0.7 | 53.7 | 8.7 | 22.0 | 9.1 | 8.0 | 50.9 | 0.1279 | 90.0 |
| Heather | 65.7 | 14.9 | 52.7 | 0.8 | 54.0 | 10.0 | 24.7 | 10.1 | 7.9 | 50.1 | 0.1266 | 114.1 |
| Hercule | 135.9 | 15.5 | 116.9 | 1.2 | 54.3 | 5.0 | 15.0 | 12.3 | 10.2 | 78.0 | 0.1259 | 158.1 |
| Jari | 75.8 | 18.3 | 58.5 | 1.0 | 54.7 | 8.0 | 27.3 | 11.0 | 10.0 | 75.2 | 0.1300 | 129.4 |
| Jutra | 42.1 | 10.3 | 27.8 | 0.7 | 54.7 | 6.3 | 20.3 | 8.8 | 8.6 | 58.1 | 0.1287 | 80.9 |
| KG-41 | 105.7 | 10.7 | 91.5 | 1.1 | 55.0 | 9.3 | 20.7 | 11.7 | 7.5 | 49.5 | 0.1255 | 148.3 |
| KORADA | 79.6 | 14.9 | 65.7 | 1.0 | 55.7 | 4.3 | 17.0 | 11.1 | 10.8 | 83.1 | 0.1304 | 135.0 |
| Kamichis | 209.9 | 11.3 | 193.3 | 1.4 | 56.3 | 7.0 | 19.3 | 13.1 | 10.2 | 79.8 | 0.1284 | 176.6 |
| Katrina | 113.4 | 13.3 | 96.7 | 1.2 | 56.7 | 7.3 | 21.0 | 12.0 | 10.1 | 75.2 | 0.1291 | 151.8 |
| Krios | 72.8 | 13.5 | 64.2 | 0.9 | 56.7 | 10.0 | 21.0 | 10.7 | 9.8 | 71.8 | 0.1276 | 124.7 |
| Lotus | 84.6 | 12.9 | 72.7 | 1.0 | 56.7 | 9.3 | 22.0 | 11.2 | 8.0 | 50.5 | 0.1274 | 140.4 |
| Madison | 44.7 | 13.3 | 27.5 | 0.7 | 57.0 | 2.3 | 13.7 | 9.1 | 8.5 | 57.4 | 0.1297 | 86.5 |
| Madoc | 36.8 | 11.8 | 21.0 | 0.6 | 57.0 | 7.7 | 23.0 | 8.3 | 6.9 | 39.7 | 0.1287 | 69.1 |
| Mandarin | 184.6 | 15.4 | 164.0 | 1.4 | 57.3 | 7.3 | 18.7 | 13.1 | 9.9 | 74.1 | 0.1276 | 175.8 |
| MapleAmbr | 95.5 | 12.9 | 79.4 | 1.1 | 57.3 | 5.7 | 19.7 | 11.4 | 9.4 | 65.2 | 0.1276 | 143.4 |
| MapleArrow | 325.0 | 11.5 | 308.3 | 1.7 | 57.3 | 3.3 | 13.3 | 13.7 | 9.4 | 66.5 | 0.1283 | 184.7 |
| MapleBelle | 55.5 | 13.0 | 38.7 | 0.8 | 57.3 | 10.3 | 28.0 | 9.7 | 10.0 | 74.2 | 0.1287 | 100.9 |
| MapleDonovan | 165.9 | 13.4 | 149.0 | 1.3 | 57.7 | 8.3 | 24.6 | 12.9 | 9.8 | 71.9 | 0.1275 | 170.4 |
| MapleGlen | 129.4 | 14.2 | 111.7 | 1.2 | 58.3 | 9.0 | 21.7 | 12.2 | 10.9 | 84.6 | 0.1285 | 155.5 |
| MapleIsle | 59.6 | 13.9 | 51.5 | 0.8 | 58.7 | 7.7 | 18.3 | 9.8 | 6.9 | 41.8 | 0.1279 | 104.1 |
| MaplePresto | 147.6 | 16.1 | 128.1 | 1.3 | 59.0 | 4.7 | 22.3 | 12.5 | 8.2 | 54.3 | 0.1270 | 164.2 |
| MapleRidge | 82.1 | 14.2 | 68.9 | 1.0 | 59.7 | 5.3 | 13.3 | 11.2 | 8.9 | 60.8 | 0.1279 | 139.2 |
| Mario | 119.3 | 15.9 | 100.0 | 1.2 | 59.7 | 15.0 | 28.3 | 12.1 | 9.0 | 61.9 | 0.1277 | 153.1 |
| McCall | 71.3 | 16.5 | 59.6 | 0.9 | 60.3 | 5.0 | 33.7 | 10.6 | 11.0 | 84.8 | 0.1289 | 121.2 |
| Misty | 79.1 | 15.6 | 64.4 | 1.0 | 60.3 | 8.7 | 19.7 | 11.1 | 11.2 | 91.5 | 0.1276 | 132.2 |
| Naya | 109.7 | 13.5 | 92.8 | 1.2 | 60.3 | 1.3 | 19.3 | 12.0 | 9.9 | 73.7 | 0.1297 | 149.2 |
| OAC1-26 | 52.4 | 11.4 | 38.0 | 0.8 | 60.7 | 6.3 | 27.7 | 9.4 | 7.1 | 44.0 | 0.1291 | 98.6 |
| OAC7-23C | 46.4 | 14.3 | 29.1 | 0.7 | 60.7 | 5.7 | 19.0 | 9.1 | 7.9 | 49.8 | 0.1271 | 89.7 |
| OAC7-26C | 459.7 | 16.1 | 438.4 | 2.0 | 60.7 | 1.7 | 17.3 | 14.4 | 9.8 | 73.2 | 0.1268 | 193.3 |
| OAC7-3C | 55.1 | 11.6 | 39.7 | 0.8 | 60.7 | 15.0 | 32.3 | 9.6 | 7.5 | 47.7 | 0.1286 | 99.7 |
| OAC7-48C | 59.0 | 10.3 | 54.6 | 0.8 | 61.3 | 7.7 | 18.7 | 9.8 | 9.6 | 68.6 | 0.1272 | 103.2 |
| OAC7-4C | 178.6 | 14.6 | 158.7 | 1.3 | 61.3 | 5.3 | 19.0 | 12.9 | 9.0 | 61.2 | 0.1275 | 172.8 |
| OAC7-6C | 28.3 | 10.2 | 15.4 | 0.5 | 61.3 | 2.0 | 12.3 | 8.1 | 6.8 | 39.2 | 0.1176 | 63.2 |
| OAC8-11C | 64.6 | 16.3 | 54.1 | 0.8 | 61.3 | 8.7 | 19.5 | 10.0 | 9.1 | 62.4 | 0.1266 | 111.5 |
| OAC8-21C | 394.4 | 12.6 | 376.5 | 1.7 | 61.7 | 3.0 | 17.7 | 13.7 | 11.9 | 95.9 | 0.1275 | 187.1 |
| OAC8-22C | 72.0 | 10.2 | 66.6 | 0.9 | 61.7 | 2.0 | 13.3 | 10.7 | 7.5 | 48.5 | 0.1302 | 124.4 |
| OAC9-17C | 49.8 | 11.7 | 35.0 | 0.7 | 61.7 | 6.0 | 14.0 | 9.3 | 8.5 | 57.7 | 0.1294 | 96.8 |
| OAC9-22C | 79.2 | 13.2 | 66.9 | 1.0 | 62.7 | 2.7 | 14.3 | 11.1 | 9.7 | 70.3 | 0.1273 | 132.5 |
| OAC9-35C | 42.3 | 14.5 | 23.8 | 0.7 | 63.0 | 10.7 | 27.7 | 8.9 | 8.1 | 51.5 | 0.1286 | 82.5 |
| OAC9-44C | 71.6 | 14.2 | 62.3 | 0.9 | 63.0 | 13.0 | 15.0 | 10.6 | 7.6 | 49.6 | 0.1289 | 123.6 |
| OAC9-48C | 87.9 | 17.7 | 66.9 | 1.0 | 63.3 | 1.7 | 19.7 | 11.3 | 10.0 | 74.2 | 0.1264 | 142.7 |
| OACAyton | 74.4 | 16.3 | 62.9 | 0.9 | 63.3 | 13.3 | 28.2 | 10.8 | 10.3 | 80.2 | 0.1318 | 125.0 |
| OACBayfield | 521.2 | 12.6 | 503.4 | 2.0 | 63.7 | 5.0 | 17.0 | 14.5 | 8.7 | 59.7 | 0.1277 | 202.6 |
| OACChampion | 76.1 | 13.7 | 63.3 | 1.0 | 64.0 | 5.7 | 19.7 | 11.0 | 11.0 | 87.0 | 0.1288 | 130.2 |
| OACKent | 51.6 | 12.3 | 36.3 | 0.7 | 64.7 | 10.7 | 30.7 | 9.3 | 10.3 | 80.5 | 0.1285 | 97.3 |
| OACLakeview | 72.8 | 14.2 | 63.4 | 0.9 | 65.7 | 6.3 | 30.7 | 10.8 | 9.7 | 70.6 | 0.1271 | 124.7 |
| OACMorris | 56.2 | 13.9 | 38.6 | 0.8 | 65.7 | 10.3 | 22.7 | 9.7 | 8.9 | 61.2 | 0.1285 | 101.4 |
| OACOxford | 65.0 | 14.3 | 52.6 | 0.8 | 65.7 | 5.7 | 15.7 | 10.1 | 6.9 | 41.4 | 0.1272 | 113.6 |
| OACPrudence | 214.9 | 13.1 | 196.6 | 1.4 | 66.0 | 4.7 | 14.3 | 13.3 | 9.9 | 73.4 | 0.1276 | 179.5 |
| OACWallace | 108.0 | 11.6 | 93.0 | 1.2 | 66.0 | 6.3 | 21.0 | 12.0 | 11.6 | 94.6 | 0.1286 | 148.8 |
| OT05-20 | 75.1 | 13.4 | 66.6 | 1.0 | 66.3 | 5.0 | 18.3 | 10.9 | 8.7 | 58.8 | 0.1289 | 125.6 |
| OT09-03 | 55.6 | 10.3 | 41.5 | 0.8 | 66.3 | 3.0 | 17.0 | 9.7 | 6.6 | 35.2 | 0.1265 | 100.9 |
| OT10-02 | 60.7 | 13.6 | 53.0 | 0.8 | 66.7 | 7.3 | 18.0 | 9.8 | 9.6 | 70.2 | 0.1291 | 105.0 |
| OT11-01 | 53.9 | 11.2 | 39.0 | 0.8 | 66.7 | 4.0 | 13.3 | 9.6 | 8.2 | 53.2 | 0.1276 | 99.3 |
| OT11-02 | 61.1 | 16.7 | 50.2 | 0.8 | 67.3 | 7.3 | 21.3 | 9.9 | 7.5 | 47.7 | 0.1288 | 105.7 |
| OT11-03 | 133.8 | 10.7 | 119.6 | 1.2 | 67.7 | 5.0 | 17.0 | 12.2 | 10.4 | 81.1 | 0.1294 | 156.5 |
| OT11-09 | 53.5 | 15.1 | 34.6 | 0.8 | 68.0 | 1.7 | 9.7 | 9.4 | 6.2 | 34.6 | 0.1267 | 98.8 |
| OT94-47 | 449.7 | 11.0 | 433.5 | 1.9 | 68.0 | 3.7 | 19.0 | 14.3 | 12.1 | 98.4 | 0.1276 | 188.8 |
| Ohgata | 80.6 | 14.9 | 66.7 | 1.0 | 68.0 | 8.3 | 27.0 | 11.1 | 9.5 | 68.3 | 0.1281 | 137.2 |
| PRO25-53 | 249.9 | 14.5 | 230.1 | 1.4 | 68.3 | 7.7 | 21.7 | 13.3 | 9.6 | 69.0 | 0.1284 | 181.3 |
| PS36 | 54.5 | 14.5 | 36.3 | 0.8 | 68.7 | 10.7 | 26.7 | 9.6 | 9.3 | 64.2 | 0.1297 | 99.6 |
| PS44 | 104.4 | 18.4 | 82.8 | 1.1 | 69.7 | 3.7 | 13.0 | 11.7 | 10.5 | 81.3 | 0.1276 | 147.8 |
| Perth | 84.9 | 14.2 | 71.7 | 1.0 | 69.7 | 7.3 | 26.7 | 11.3 | 7.0 | 43.2 | 0.1264 | 141.0 |
| Phoenix | 150.9 | 19.6 | 127.8 | 1.3 | 71.7 | 4.3 | 13.7 | 12.6 | 10.9 | 84.1 | 0.1263 | 164.3 |
| Proteus | 101.5 | 12.8 | 85.4 | 1.1 | 73.7 | 7.0 | 32.0 | 11.5 | 12.0 | 98.3 | 0.1281 | 144.8 |
| Purdy | 67.7 | 11.7 | 57.9 | 0.9 | 74.3 | 6.3 | 13.7 | 10.3 | 7.4 | 46.4 | 0.1281 | 117.3 |
| Roland | 44.6 | 12.9 | 27.7 | 0.7 | 74.7 | 4.7 | 13.3 | 9.0 | 7.0 | 42.1 | 0.1282 | 86.1 |
| S03-W4 | 87.3 | 14.6 | 69.4 | 1.0 | 75.0 | 3.0 | 16.7 | 11.3 | 9.4 | 67.7 | 0.1197 | 142.6 |
| S05-T6 | 103.0 | 14.5 | 85.3 | 1.1 | 75.3 | 6.7 | 21.0 | 11.5 | 9.3 | 65.0 | 0.1287 | 144.9 |
| S12-A5 | 66.0 | 18.6 | 49.2 | 0.9 | 76.0 | 4.7 | 16.3 | 10.2 | 8.3 | 55.6 | 0.1267 | 114.6 |
| S14-P6 | 71.9 | 15.9 | 60.9 | 0.9 | 76.0 | 6.3 | 16.0 | 10.7 | 10.1 | 77.0 | 0.1274 | 123.7 |
| SECAN7-27 | 81.1 | 16.4 | 65.7 | 1.0 | 76.3 | 8.3 | 20.0 | 11.1 | 10.4 | 80.6 | 0.1318 | 138.9 |
| SECAN7-49 | 103.4 | 10.2 | 90.0 | 1.1 | 76.3 | 8.3 | 24.3 | 11.5 | 9.7 | 71.6 | 0.1298 | 145.3 |
| SECAN8-10 | 41.9 | 10.7 | 27.2 | 0.7 | 78.3 | 11.0 | 28.3 | 8.8 | 6.6 | 37.0 | 0.1284 | 78.9 |
| SECAN8-17 | 71.0 | 11.5 | 61.4 | 0.9 | 79.3 | 7.0 | 20.3 | 10.6 | 8.2 | 52.8 | 0.1274 | 120.0 |
| SECAN8-1C | 75.2 | 16.4 | 63.6 | 1.0 | 79.3 | 9.0 | 26.0 | 10.9 | 9.0 | 62.1 | 0.1287 | 129.3 |
| SECAN8-24 | 182.9 | 14.8 | 162.8 | 1.3 | 79.7 | 5.0 | 20.0 | 13.0 | 7.3 | 44.8 | 0.1294 | 175.4 |
| SECAN9-38 | 132.6 | 16.6 | 112.6 | 1.2 | 80.0 | 3.0 | 13.3 | 12.2 | 9.4 | 66.4 | 0.1266 | 155.6 |
| Saska | 287.8 | 13.3 | 269.2 | 1.5 | 81.3 | 6.0 | 20.7 | 13.4 | 9.6 | 68.4 | 0.1278 | 182.9 |
| Stratford | 57.2 | 13.8 | 39.7 | 0.8 | 83.0 | 5.3 | 19.0 | 9.7 | 8.1 | 51.3 | 0.1293 | 101.6 |
| Supra | 122.7 | 11.1 | 108.1 | 1.2 | 83.7 | 10.3 | 25.7 | 12.1 | 9.0 | 62.0 | 0.1291 | 154.5 |
| Toki | 159.8 | 13.5 | 142.9 | 1.3 | 84.0 | 4.7 | 16.3 | 12.8 | 9.5 | 67.8 | 0.1286 | 168.4 |
| Tundra | 97.0 | 12.7 | 81.1 | 1.1 | 84.3 | 5.3 | 13.0 | 11.5 | 13.8 | 103.1 | 0.1272 | 144.8 |
| Venus | 289.5 | 18.5 | 265.7 | 1.6 | 84.3 | 7.3 | 26.7 | 13.6 | 6.8 | 37.9 | 0.1280 | 183.0 |
| Victoria | 41.5 | 10.4 | 27.1 | 0.7 | 85.7 | 5.3 | 20.3 | 8.7 | 5.8 | 32.0 | 0.1288 | 74.4 |
| Walton | 58.2 | 15.4 | 48.6 | 0.8 | 87.3 | 3.0 | 16.3 | 9.8 | 10.1 | 76.8 | 0.1258 | 102.9 |
| Woodstock | 530.4 | 11.5 | 513.6 | 2.2 | 122.7 | 7.3 | 32.0 | 15.5 | 12.3 | 101.2 | 0.1284 | 207.7 |

**Supplementary Table 2**. Composition of nutrient solution used to fertilize soybean plants.

| **Compound** | **Concentration of stock solution**  **(g L^-1^)** | **Quantity in the final solution**  **(mg L^-1^)** | **Volume of stock solution per L in final solution (ml L^-1^)** |
| --- | --- | --- | --- |
| **KCl** | 745.52 | 745.50 | 10 |
| **KNO_3_** | 101.10 | 1011.10 | 10 |
| **MgSO_4_.7H_2_O** | 246.48 | 492.96 | 2 |
| **K_2_HPO_4_** | 174.18 | 174.18 | 1 |
| **KH_2_PO_4_** | 136.09 | 136.09 | 1 |
| **CaCl_2_.2H_2_O** | 147.01 | 147.01 | 1 |
| **Fe-Sequestrate 330** | 20.00 | 20.00 | 1 |
| **H_3_BO_3_** | 1.00 |  | 1 |
| **MnCl_2_.4H_2_O** | 1.00 |  | 1 |
| **ZnSO_4_.7H_2_O** | 0.58 |  | 1 |
| **CuSO_4_.5H_2_O** | 0.13 |  | 1 |
| **Na_2_MoO_4_.2H_2_O** | 0.10 |  | 1 |
| **CoCl_2_.6H_2_O** | 1 ml of solution (1x10^-3^ g / L) |  | 1 |

**Supplementary Table 3**: Detailed description of the 12 traits captured by ARIA (Pace et al., 2014).

| **Trait name** | **Abbreviation** | **Description** |
| --- | --- | --- |
| Total length of roots | TLR | The cumulative length of all the roots in centimeters |
| Length of primary root | LPR | The length of primary root in centimeters |
| Length of secondary roots | LSR | The cumulative length of all secondary roots in centimeters |
| Distribution of total root length | DTLR | Length of roots in the upper third of the root system / TLR |
| Total number of roots | TNR | The number root number in whole root system |
| Median | Med | The result of a vertical line sweep in which the number of roots that crossed a horizontal line was estimated, and then the median of all values for the extent  of the network was calculated. |
| Maximum | Max | After sorting the number of roots crossing a horizontal line from smallest to largest, the maximum number is considered to be the 84th-percentile value (one standard deviation). |
| Depth of root system | DRS | The maximum vertical distance reached by the root system |
| Width of root system | WRS | The maximum horizontal width of the whole root system architecture |
| Surface of root system | SRS | The area of the convex hull that enclosed the entire root image |
| Diameter of primary root | DR | The value of the primary root width estimation |
| Surface area of primary root | SAR | The area of the primary root |

**Supplementary Table 4**. Summary of variance analyses for all RSA-related traits. TLR : total length of roots, LPR : length of primary root, LSR : length of secondary roots, DTLR : distribution of total root length, TNR : total number of roots, Med : median number of roots, Max : maximum number of roots, DRS : depth of root system, WRS : width of root system, SRS : surface of root system, DR : diameter of primary root, SAR : surface area of primary root. Root.

Signif. codes: ‘***’ 0.001 ‘**’ 0.01 ‘*’ 0.05

**(A) ANOVA for TLR**

|  | Df | Sum Sq | Mean Sq | F value | Pr(>F) |
| --- | --- | --- | --- | --- | --- |
| Genotype | 136 | 3271645 | 24056.2 | 4440.437 | <2e-16 *** |
| Rep | 2 | 264 | 132.1 | 24.386 | 1.814 |
| Residuals | 272 | 1474 | 5.4 |  |  |

**(B) ANOVA for LPR**

|  | Df | Sum Sq | Mean Sq | F value | Pr(>F) |
| --- | --- | --- | --- | --- | --- |
| Genotype | 136 | 2156.0044 | 15.8530 | 42.996 | <2.2e-16*** |
| Rep | 2 | 86.9637 | 43.4818 | 118.21 | < 2.2e-16 *** |
| Residuals | 272 | 100.055 | 0.3678 |  |  |

**(C) ANOVA for LSR**

|  | Df | Sum Sq | Mean Sq | F value | Pr(>F) |
| --- | --- | --- | --- | --- | --- |
| Genotype | 136 | 3.168E+06 | 2.329E+04 | 23016.18 | <2.2e-16*** |
| Rep | 2 | 4.469E+01 | 2.234E+01 | 22.08 | 1.287e-09 *** |
| Residuals | 272 | 2.753E+02 | 1.012E+00 |  |  |

**(D) ANOVA for DTLR**

|  | Df | Sum Sq | Mean Sq | F value | Pr(>F) |
| --- | --- | --- | --- | --- | --- |
| Genotype | 136 | 38.430885 | 0.282580 | 44.99 | <2.2e-16 *** |
| Rep | 2 | 0.364535 | 0.182268 | 29.02 | 2.193e-12 *** |
| Residuals | 272 | 1.708520 | 0.006281 |  |  |

**(E) ANOVA for TNR**

|  | Df | Sum Sq | Mean Sq | F value | Pr(>F) |
| --- | --- | --- | --- | --- | --- |
| Genotype | 136 | 78705 | 578.72 | 351.3607 | <2.e-16 *** |
| Rep | 2 | 7 | 3.49 | 2.1206 | 0.1219 |
| Residuals | 272 | 448 | 1.65 |  |  |

**(F) ANOVA for Med**

|  | Df | Sum Sq | Mean Sq | F value | Pr(>F) |
| --- | --- | --- | --- | --- | --- |
| Genotype | 136 | 3813.7578 | 28.0423 | 167.35 | <2e-16 *** |
| Rep | 2 | 136 | 30.1200 | 179.75 | <2e-16 *** |
| Residuals | 272 | 45.5771 | 0.1676 |  |  |

**(G) ANOVA for Max**

|  | Df | Sum Sq | Mean Sq | F value | Pr(>F) |
| --- | --- | --- | --- | --- | --- |
| Genotype | 136 | 11163.5 | 82.085 | 2.7283e+30 | < 2.2e-16 *** |
| Rep | 2 | 376.3 | 188.160 | 6.2539e+30 | < 2.2e-16 *** |
| Residuals | 272 | 0.0 | 0.000 |  |  |

**(H) ANOVA for DRS**

|  | Df | Sum Sq | Mean Sq | F value | Pr(>F) |
| --- | --- | --- | --- | --- | --- |
| Genotype | 136 | 954.0776 | 7.0153 | 22.57 | <2.2e-16 *** |
| Rep | 2 | 15.2040 | 7.6020 | 24.46 | <2.2e-16 *** |
| Residuals | 272 | 84.5512 | 0.3108 |  |  |

**(I) ANOVA for WRS**

|  | Df | Sum Sq | Mean Sq | F value | Pr(>F) |
| --- | --- | --- | --- | --- | --- |
| Genotype | 136 | 980.1339 | 7.2069 | 9.36 | <2.e-16*** |
| Rep | 2 | 27.0859 | 13.5430 | 17.58 | 6.572e-08*** |
| Residuals | 272 | 209.5171 | 0.7703 |  |  |

**(J) ANOVA for SRS**

|  | Df | Sum Sq | Mean Sq | F value | Pr(>F) |
| --- | --- | --- | --- | --- | --- |
| Genotype | 136 | 47509.7 | 349.3 | 1.19 | 0.120 |
| Rep | 2 | 0.7 | 0.3 | 0.00 | 0.9989 |
| Residuals | 272 | 80079.8 | 294.4 |  |  |

**(K) ANOVA for DR**

|  | Df | Sum Sq | Mean Sq | F value | Pr(>F) |
| --- | --- | --- | --- | --- | --- |
| Genotype | 136 | 0.00126647 | 9.3123e-06 | 335.583 | <2.2e-16 *** |
| Rep | 2 | 0.00000269 | 1.3462e-06 | 48.512 | <2.2e-16 *** |
| Residuals | 272 | 0.00000755 | 2.7700e-08 |  |  |

**(L) ANOVA for SAR**

|  | Df | Sum Sq | Mean Sq | F value | Pr(>F) |
| --- | --- | --- | --- | --- | --- |
| Genotype | 136 | 483216.473 | 3553.062 | 1080.57 | < 2.2e-16 *** |
| Rep | 2 | 32.548 | 16.274 | 4.95 | 0.007809 ** |
| Residuals | 272 | 894.372 | 3.288 |  |  |

**Supplementary Table 5**. Shapiro-Wilk test of normality for all root system architecture traits, which tests the null hypothesis that the dependent variable is normally distributed (based on *p*-value = 0.32). Total length of roots (TLR), length of primary root (LPR), length of secondary roots (LSR), distribution of total root length (DTLR), total number of roots (TNR), median number of roots (Med), maximum number of roots (Max), depth of root system (DRS), width of root system (WRS), surface of root system (SRS), diameter of primary roots (DR), surface area of primary root (SAR)

| **Trait** | **W** | **P-value** |
| --- | --- | --- |
| **TLR** | 0.64 | < 2.2e-16 |
| **LPR** | 0.99 | 0.52 |
| **LSR** | 0.63 | < 2.2e-16 |
| **DTLR** | 0.92 | 3.3e-07 |
| **TNR** | 0.97 | 0.002 |
| **Med** | 0.96 | 0.002 |
| **Max** | 0.97 | 0.003 |
| **DRS** | 0.98 | 0.042 |
| **WRS** | 0.98 | 0.21 |
| **SRS** | 0.99 | 0.52 |
| **DR** | 0.84 | 8.2e-11 |
| **SAR** | 0.99 | 0.79 |

**Supplementary Table 6.** Details of quantitative trait loci (QTL) associated with total length of roots (TLR) and diameter of roots (DR) related to root system architecture among a core collection of 137 soybean lines.

| **Trait** | **Chr** | **QTL ID** | **MSS**  **Position (bp)** | **QTL**  **size (kb)** | **Significant region** | |
| --- | --- | --- | --- | --- | --- | --- |
|  |  |  |  |  | ***Start*** | ***End*** |
| TLR | 01 | *qTLR1* | 39,473,722 | 45 | 39,430,088 | 39,475,387 |
|  | 03 | *qTLR2* | 11,872,785 | 207 | 11,792,295 | 11,999,126 |
|  | 03 | *qTLR3* | 26,421,602 | 6 | 26,362,442 | 26,426,995 |
|  | 10 | *qTLR4* | 33,249,968 | 85 | 33,166,905 | 33,252,259 |
|  | 18 | *qTLR5* | 15,820,143 | 244 | 15,787,915 | 16,031,981 |
|  | 19 | *qTLR6* | 12,124,915 | 13 | 12,111,710 | 12,124,926 |
| DR | 06 | *qDR1* | 3,828,365 | 30 | 3,806,192 | 3,836,771 |
|  | 07 | *qDR2* | 8,991,589 | 1.8 | 8,990,295 | 8,992,090 |
|  | 13 | *qDR3* | 5,944,486 | 102 | 5,842,404 | 5,944,583 |
|  | 18 | *qDR4* | 33,584,142 | 425 | 33,161,353 | 33,587,050 |

**Supplementary Table 7**. Full list of genes residing in the significant haplotype blocks / quantitative trait locus regions associated with total length of roots (TLR) and diameter of roots (DR) traits related to root system architecture among a core collection of 137 soybean lines.

| **Trait** | **Chr** | **QTL** | **Gene name** | **Gene start** | **Gene end** | **Annotation in SoyBase** |
| --- | --- | --- | --- | --- | --- | --- |
| TLR | 01 | *qTLR1* | Glyma.01g114100 | 39,440,338 | 39,440,694 | Mitogen-activated protein kinases (MAPK) cascade |
|  | 03 | *qTLR2* | Glyma.06g035000 | 11,896,538 | 11,909,845 | Scarecrow-like protein 9-like / Short-root (putative transcription factor GRAS) / Control of root radial patterning and root growth |
|  | 03 | *qTLR2* | There were no gene identified | | | |
|  | 10 | *qTLR4* | Glyma.10g125900 | 33,171,187 | 33,171,431 | Auxin-induced protein |
|  |  |  | Glyma.10g126000 | 33,176,626 | 33,177,099 | S locus-related glycoprotein 1 binding pollen coat protein (SLR1-BP) |
|  | 18 | *qTLR5* | Glyma.18g123100 | 15,786,606 | 15,787,915 | Sterol regulatory element-binding protein |
|  |  |  | Glyma.18g123200 | 15,832,903 | 15,834,106 | Unknown |
|  | 19 | *qTLR6* | Glyma.19g060700 | 12,112,589 | 12,113,599 | MYB domain proteins act as DNA-binding transcription factors/ Regulation root hair cell differentiation |
| DR | 06 | *qDR1* | Glyma.06g050200 | 3,813,696 | 3,818,031 | Cell wall / Extracellular region |
|  |  |  | Glyma.06g050300 | 3,823,010 | 3,824,607 | Zinc finger C-x8-C-x5-C-x3-H type (and similar) (zf-CCCH) |
|  |  |  | Glyma.06g050400 | 3,833,600 | 3,836,739 | Protein of Unknown Function (DUF239) |
|  | 07 | *qDR2* | Glyma.07g096000 | 8,990,337 | 8,991,434 | Probable receptor-like protein kinase/GUB_WAK_bind/Key regulator controlling root hairs development, significantly increases the overall surface area/diameter of a root |
|  | 13 | *qDR3* | Glyma.13g026400 | 5,845,494 | 5,847,686 | Clathrin coat assembly protein AP180 |
|  |  |  | Glyma.13g026500 | 5,866,198 | 5,866,734 | Unknown |
|  |  |  | Glyma.13g026600 | 5,881,744 | 5,896,487 | Nuclease-ascorbate transporter1 |
|  | 18 | *qDR4* | Glyma.18g155200 | 33,270,053 | 33,274,748 | U-BOX domain-containing protein 9 |

**Supplementary Table 8.** Comparing detected QTL regions associated for TLR and LSR, DTLR, DRS which were very highly and significantly correlated each other. Length of secondary roots (LSR), distribution of total root length (DTLR), depth of root system (DRS), surface area of primary root (SAR)

| **Trait** | **QTL regions significantly associated with TLR** | | | | | |
| --- | --- | --- | --- | --- | --- | --- |
|  | *qTLR1* | *qTLR2* | *qTLR3* | *qTLR4* | *qTLR5* | *qTLR6* |
| LSR | ✓ | ✓ | ✓ | ✓ |  | ✓ |
| DTLR | ✓ | ✓ |  | ✓ | ✓ | ✓ |
| DRS |  | ✓ | ✓ | ✓ |  | ✓ |
| SAR | ✓ | ✓ |  | ✓ |  |  |

**Supplementary Table 9**: Significantly associated SNPs for RSA-related traits highly correlated with the Total length of roots (TLR). The same SNP (or one very near) is found to show a significant association. Length of secondary roots (LSR), distribution of total root length (DTLR), depth of root system (DRS), surface area of primary root (SAR).

| **Chr** | **Position of MSS**  **(FDR value)** | **QTL ID** | **LSR** | **DTLR** | **DR**S | **SAR** |
| --- | --- | --- | --- | --- | --- | --- |
| **01** | 39,473,722  (1.3e-2) | *qTLR1* | Same SNP  (1.2e-2) | Same SNP  (1.1e-2) | 39,473,800  (1.6e-2) | Same SNP  (1.0e-2) |
| **03** | 11,872,785  (2.2e-10) | *QTLR2* | Same SNP (1.9e-10) | Same SNP (2.5e-10) | Same SNP  (2.3e-10) | Same SNP  (2.7e-10) |
| **03** | 26,421,602  (7.2e-4) | *qTLR3* | Same SNP  (7.9e-4) | 26,422,727  (7.5e-4) | Same SNP  (8.0e-4) | 26,419,385  (7.5e-4) |
| **10** | 33,249,968  (1.3e-2) | *qTLR4* | Same SNP  (1.6e-2) | Same SNP  (1.9e-2) | Same SNP  (1.6e-2) | Same SNP  (1.5e-2) |
| **18** | 15,820,143  (1.1e-2) | *qTLR5* | 15,821,935  (1.3e-2) | Same SNP  (1.2e-2) | 15819954  (1.8e-2) | 15,820,108  (1.5e-2) |
| **19** | 12,124,915  (9.1e-5) | *qTLR6* | Same SNP  (9.3e-5) | Same SNP  (8.9e-5) | Same SNP  (9.0e-5) | 12,124,804  (9.4e-5) |
